# The autonomic nervous system as a piece of the mechanistic puzzle linking sleep and atrial fibrillation

**DOI:** 10.1007/s10840-022-01290-1

**Published:** 2022-07-18

**Authors:** Christian Meyer, Ann-Kathrin Kahle

**Affiliations:** 1 Division of Cardiology, Angiology, Intensive Care Medicine, cNEP, Cardiac Neuro- and Electrophysiology Research Consortium, EVK Düsseldorf, Kirchfeldstrasse 40, 40217 Düsseldorf, Germany; 2 Institute of Neural and Sensory Physiology, cNEP, Cardiac Neuro- and Electrophysiology Research Consortium, Heinrich Heine University Düsseldorf, Medical Faculty, Universitätsstrasse 1, 40225 Düsseldorf, Germany; 3 Department of Cardiology, Pulmonology and Vascular Medicine, Medical Faculty, University Hospital Düsseldorf, Moorenstrasse 5, 40225 Düsseldorf, Germany

The autonomic nervous system is well known to play an integral role in the development and progression of atrial fibrillation (AF) as well as in sleep and circadian rhythm pathophysiology [1]. More than a century ago, vagal stimulation was found to be an adequate method for obtaining AF [2]. Following the pioneering clinical observations by Coumel et al. [3] in the 1970s, alteration of autonomic tone assessed by heart rate variability (HRV) immediately before the onset of paroxysmal AF (PAF) has been described [4].

Obstructive sleep apnea (OSA) is the most common sleep disorder observed in about 34% and 17% of middle-aged men and women [5]. It is well known that OSA and AF share common risk factors such as obesity, increasing age, male sex, hypertension, and heart failure. Screening for the often underdiagnosed OSA in patients with recurrent AF after either cardioversion or ablation is recommended by the American Heart Association [5]. However, even though OSA and AF often coexist, their relationship has been frequently discussed, with several potential underlying mechanisms being suggested [1]. Chronic recurrence of intermittent hypoxia with acute alteration in intrathoracic pressure may lead to long-term atrial remodeling altering atrial electrophysiology [5]. Long pauses and bradycardia are common in patients with OSA, and those episodes might be related to increased parasympathetic activity [1]. Vagal effects of shortening the action potential and refractory period are further recognized to be nonhomogeneous in the atrial wall. At the same time, sympathetic activation in patients with OSA is well known. This is important since experimental [6] and first clinical evidence [7] suggests that autonomic coactivation proceeds the onset of AF. These effects of the sympathetic and parasympathetic autonomic nervous system potentially resulting in a predisposition to AF may be assessed by analysis of HRV [4] and autonomic bedside testing [7, 8].

In this issue of JICE, Mohammadieh et al. [9] present first evidence on cardiac autonomic function measured by HRV in PAF patients with and without OSA. A total of 101 patients with a history of AF were enrolled in this prospective dual-center study and underwent in-laboratory polysomnography including Holter analysis. Overall, HRV parameters did not differ between PAF patients with and without OSA. As indicated by frequency-domain analysis of HRV, during non-rapid eye movement sleep, PAF patients with OSA showed increased cardiac parasympathetic activity (increased high-frequency power) and reduced cardiac sympathetic activity (reduced low-frequency power and ratio of low-/high-frequency power) compared to patients without OSA. There were no differences during rapid eye movement sleep. Interestingly, Mohammadieh et al. found an increase in parasympathetic activity in PAF patients with OSA, suggesting vagal predominance as the main contributor to AF induction in this population. Although puzzling, these findings highlight the importance of the autonomic nervous system linking sleep and atrial fibrillation.

We congratulate the authors on the first study of HRV in PAF patients with and without OSA providing interesting findings on cardiac autonomic function in this population. The authors present relevant hypothesis-generating data. The present investigations also highlight some challenges which are related to such study designs and the still somehow limited methods to evaluate the activity of the autonomic nervous system in clinical scenarios.

In the past, the autonomic mechanisms preceding the onset of AF have been widely studied by HRV in various patient cohorts leading to partly diverging results (Table 1). Whereas some groups described predominance of either the sympathetic or parasympathetic nervous system, more recently, others found evidence for autonomic coactivation linked to heart rate dynamics in patients with PAF [7]. In a greater detail, observation of selected time points before AF onset indicated consecutive autonomic changes, with an initial increase in sympathetic followed by parasympathetic activity. Classification of AF episodes into those occurring at day- or nighttime has demonstrated that especially nocturnal AF might be associated with an increase in parasympathetic activity without any change in sympathetic tone (Table 1). In the here presented work [9], besides the noted parasympathetic predominance, reduced sympathetic activity during non-rapid eye movement sleep has been reported in patients with OSA compared to those without. This observation might be explained and further reinforced by a ceiling effect driven by a higher intrinsic sympathetic tone based on recurrent apneic events in this population.

**Table 1:** Changes in heart rate variability in patients with atrial fibrillation

Study	Study population	Duration of HRV analysis	Frequency-domain analysis	Time-domain analysis	Non-linear analysis	Conclusion
Dimmer et al., Am J Cardiol. 1998	Pts with AF after coronary artery bypass grafting vs controls	1-h period before AF onset divided into 4 intervals of 15 min each	↔ HF, ↔ LF, ↓ L/H ratio during 60–30 min before onset in AF pts vs controls, ↑ L/H ratio in AF pts over time, ↔ L/H ratio in controls over time	↑ SDNN during the last 15 min before onset in AF pts vs controls	-	↑ Sympathetic and ↓ parasympathetic
Herweg et al., Am J Cardiol. 1998	Pts with daytime vs nighttime AF episodes	Time points at 5, 10, and 20 min before AF onset	• Overall: ↔ HR, ↔ HF, ↔ LF, ↔ L/H ratio, ↑ change in HF norm. 10–5 min vs 20–10 min before onset, ↔ LF norm. 10–5 min vs 20–10 min before onset • Daytime episodes: ↔ HR, ↔ HF, ↔ LF, ↔ L/H ratio, ↑ change in HF norm. 10–5 min vs 20–10 min before onset, ↔ LF norm. 10–5 min vs 20–10 min before onset • Nighttime episodes: ↔ HR, ↑ HF, ↔ LF, ↓ L/H ratio 10–5 min vs 20–10 min before onset, ↑ change in HF norm. 10–5 min vs 20–10 min before onset, ↔ LF norm. 10–5 min vs 20–10 min before onset	-	-	↑ Parasympathetic, especially during nighttime
Hogue et al., Circulation. 1998	Pts with AF after coronary artery bypass grafting vs controls	1-h period before AF onset divided into 20-min intervals	↑ HR, ↔ HF, ↔ LF	↔ RR interval, ↔ SDNN, ↔ pNN50, ↔ rMSSD, ↔ SD1RR, ↔ SD2RR, ↔ SD1RR/SD2RR	↓ ApEn	↓ HR complexity
Huang et al., Int J Cardiol. 1998	Pts with PAF without or with structural heart disease	40-min period before PAF onset divided into 2-min intervals	• Pts without structural heart disease: ↑ HF, ↓ L/H ratio • Pts with structural heart disease: ↓ HF, ↑ L/H ratio	-	-	• Pts without structural heart disease: ↑ parasympathetic • Pts with structural heart disease: ↑ sympathetic
Lok and Lau, Pacing Clin Electrophysiol. 1998	Pts with PAF without structural heart disease vs controls	Average of 2 frequency bandwidths over a 24-h period	↔ HF, ↔ LF, ↔ L/H ratio	↔ SDNN, ↔ pNN50, ↔ rMSSD	-	No cardiac sympathovagal imbalance
Fioranelli et al., Pacing Clin Electrophysiol. 1999	Pts with PAF without structural heart disease	5-min period before PAF onset vs 5-min control period ≥ 1 h after AF onset	↔ HR, ↔ HF, ↔ LF, ↔ L/H ratio → Subsequent identification of 2 subgroups • Group 1: ↔ HR, ↓ HF, ↑ LF, ↑ L/H ratio • Group 2: ↓ HR, ↑ HF, ↓ LF, ↓ L/H ratio No differences according to the time of occurrence or gender	-	-	• Group 1: ↑ sympathetic • Group 2: ↑ parasympathetic
Vikman et al., Circulation. 1999	Pts with PAF without structural heart disease vs controls	2-h period before PAF onset divided into 6 intervals of 20 min each	↔ HF, ↔ LF, ↔ L/H ratio	↔ RR interval, ↔ SDNN	↓ α_1_ and ↓ ApEn over time and vs controls	↓ HRV complexity
Vikman et al., Ann Noninvasive Electrocardiol. 2001	Pts with PAF without structural heart disease • Short (< 200 s) vs long (> 200 s) PAF episodes	2-h period before PAF onset divided into 6 intervals of 20 min each	↑ HF, ↓ LF, and ↓ L/H ratio before long vs short episodes	↔ RR interval, ↓ SDNN before long vs short episodes	↓ α_1_ before long vs short episodes	↑ Parasympathetic before long episodes
Bettoni and Zimmermann, Circulation. 2002	Consecutive pts with PAF • Pts with lone AF vs with structural heart disease • Daytime vs nighttime episodes • Pts with vs without beta-blockers	6 periods: 24 h before AF onset, 1 h before AF onset, 20 min before AF onset divided into 4 intervals of 5 min each	• Overall: ↑ HF before onset, with the highest value during the 10–5 min before onset, ↑ LF from 24 h until 15 min before onset, ↓ LF from 15 min before until onset, ↔ VLF, ↔ L/H ratio • Pts with lone AF vs with structural heart disease: no differences • Nighttime vs daytime: ↑ HF and ↓ L/H ratio before nighttime vs daytime episodes, ↑ HF before nighttime episodes over time, ↔ HF before daytime episodes over time, ↑ LF during the 20 min before nighttime episodes, ↔ LF before daytime episodes • Pts with vs without beta-blockers: ↓ HF in pts with vs without beta-blockers	• Overall: ↓ RR interval from 20 min before until onset, ↔ RR interval before onset, ↑ SDNN interval from 20 min before until onset, ↓ SDNN interval 5 min vs 24 h or 1 h before onset, ↔ SDANN, ↓ SDANN interval 5 min vs 24 h or 1 h before onset, ↔ pNN50, ↔ rMSSD • Pts with lone AF vs with structural heart disease: ↓ RR interval during the 24 h before onset in pts with structural heart disease vs lone AF • Nighttime vs daytime: no differences • Pts with vs without beta-blockers: ↑ RR interval, ↑ SDNN, ↑ SDANN in pts with vs without beta-blockers	-	Initially ↑ sympathetic → ↑ parasympathetic
Amar et al., J Am Coll Cardiol. 2003	Pts with postoperative AF vs controls	2-h period before AF onset, with time points at 120, 60, 30, and 5 min before onset	• Overall: ↑ LF, ↑ HF, ↓ L/H ratio • Selected time points: ↑ HF at 120 min, 60 min, 5 min before onset, ↑ LF at 120 min, 30 min, 5 min before onset, ↓ L/H ratio at 120 min, 5 min before onset	• Overall: ↔ RR interval, ↑ SDNN, ↑ pNN50, ↑ rMSSD • Selected time points: ↑ SDNN and ↑ rMSSD at 120 min, 30 min, 5 min before onset, ↑ pNN50 at 120 min, 60 min, 30 min, 5 min before onset	-	↑ Parasympathetic and sympathetic

Dimmer et al., Europace. 2003	Pts with PAF • Accelerators vs decelerators vs stable with respect to HR variations before AF onset (changes of mean HR during the last 5 min vs mean HR of the preceding 55 min > 5%)	1-h period before AF onset divided into 2 intervals of 30 min each	↔ L/H ratio 60–30 min vs 30–0 min before onset No differences in either type	• Overall: ↔ SDNN, ↑ pNN50 30–0 min vs 60–30 min before onset, ↔ SD/RR 60–30 min vs 30–0 min before onset • Accelerators: ↑ SDNN and ↑ SD/RR 30–0 min vs 60–30 min before onset • Decelerators: no differences • Stable: no differences	-	↑ HR instability, especially in accelerators
Tomita et al., J Cardiovasc Electrophysiol. 2003	Pts with PAF without structural heart disease with day- vs nighttime episodes	1-h period before and after AF, with time points at 60, 20, and 10 min before and after onset	• Daytime: ↔ HF before and after AF, ↑ LF, and ↑ L/H ratio, with the highest value at 10 min before onset, ↓ LF, and ↓ L/H ratio 10 min after termination vs 10 min before onset, ↔ L/H ratio 10 min after termination vs 60 min before onset, ↑ L/H ratio after termination • Nighttime: ↑ HF, and LF, with the highest value at 10 min before onset, ↔ HF 10 min after termination vs 60 min before onset, ↓ HF after termination vs 10 min before onset, ↔ L/H ratio before and after AF	• Daytime: ↔ RR interval, ↔ SDNN, ↔ rMSSD • Nighttime: ↑ RR interval before onset, ↔ RR interval after termination vs 60 min before onset, ↓ RR interval 10 min after termination vs 10 min before onset, ↔ RR interval after termination, ↑ rMSSD 10 min vs 60 min before onset, ↓ rMSSD 10 min after termination vs 10 min before onset	-	• Daytime: ↑ sympathetic before onset, ↓ to basal values after termination • Nighttime: ↑ parasympathetic before onset, ↓ to basal values after termination, ↔ sympathetic
Van den Berg et al., Heart. 2003	Pts with vagal vs non-vagal AF, all without congestive heart failure	24-h period	↔ HF, ↔ LF	↑ rMSSD	-	↑ Parasympathetic tone and ↔ parasympathetic reactivity in pts with vagal AF
Lombardi et al., Eur Heart J. 2004	Consecutive pts with PAF • Type of onset: T1 (short-long-short sequence) vs T2 (sudden onset) • L/H ratio: ≥ or < 2	24-h period including a 30-min interval before AF onset, a 5-min interval after recovery of sinus rhythm, episodes ≥ 15 min were divided into the first, central, and final 5 min	• Overall: ↓ HF, ↑ LF, and ↑ L/H ratio before vs after AF • Type of onset: ↑ LF and ↓ duration of AF episodes in T1 vs T2 • L/H ratio: ↓ L/H ratio after vs before AF when ≥ 2, ↑ L/H ratio after vs before AF when < 2	• Overall: ↔ RR interval before vs after AF, ↔ RR interval, ↔ SDNN, and ↔ CV during AF • L/H ratio: ↔ RR interval before vs after AF in both groups	-	↑ Sympathetic and ↓ parasympathetic
Singh et al., Ann Noninvasive Electrocardiol. 2004	Pts with PAF vs controls	2-h period	↓ ln HF, ↓ ln LF, ↓ L/H ratio (all unadjusted), ↔ ln HF, ↔ ln LF, ↔ ln L/H ratio (all adjusted)	↓ ln SDNN (unadjusted), ↔ ln SDNN (adjusted)	-	Association between HRV and PAF is mediated by traditional risk factors
Shin et al., Circ J. 2006	Pts with PAF • Vagal type: ↑ HF, ↓ L/H ratio • Sympathetic type: ↓ HF, ↑ L/H ratio • Other type	1-h period before AF onset divided into 6 intervals of 10 min each	↑ HF, ↔ LF, ↓ L/H ratio No differences in either type	↔ pNN50, ↑ rMSSD No differences in either type	↓ ApEn, ↓ SampEn (overall and for both types) • Vagal: ↓ α_1_ • Sympathetic: ↔ α_1_	↓ Complexity of HRV
Vincenti et al., Europace. 2006	Pts with PAF vs controls	30-min period before AF onset divided into 6 intervals of 5 min each, 5-min control period ≥ 60 min after AF onset	↔ HF, ↓ LF 5–0 min before onset vs all other intervals and controls, ↓ L/H ratio 5–0 min before onset vs 10–5 min, 20–15 min, 30–25 min before onset and controls No differences according to the presence of an underlying heart disease	↑ SDNN 5–0 min before onset vs all other intervals and controls No differences according to the presence of an underlying heart disease	-	↑ Parasympathetic
Agarwal et al., J Am Coll Cardiol. 2017	Pts with AF vs controls	2 min	↓ ln HF, ↓ ln LF, ↔ ln L/H ratio	↓ ln SDNN, ↓ ln rMSSD	-	↓ HR modulation as a risk factor for AF
Nortamo et al., Heart Rhythm. 2018	Pts with PAF and coronary artery disease vs controls	24-h period	↔ HR, ↔ HF, ↓ LF, ↓ L/H ratio, ↓ VLF	↓ SDNN	↔ ApEn, ↓ DFA1	Rather ↑ sympathetic than parasympathetic
Kališnik et al., Int J Cardiol. 2019	Pts after cardiac surgery developing AF vs developing no AF	20-min period on the day before surgery	-	↑ pNN50	↓ DFA1	↑ Sympathetic and parasympathetic
Liao et al., Ann Noninvasive Electrocardiol. 2020	Pts with PAF with vs without ischemic heart disease	20-min periods before and after AF divided into 8 intervals of 5 min each	• Pts with ischemic heart disease: ↑ HF from 20 min before until onset, ↔ LF, ↑ L/H ratio from 10–5 min before onset, ↓ HF, and ↓ LF after termination, ↔ L/H ratio • Pts without ischemic heart disease: ↔ HF, ↔ LF, ↔ L/H ratio, ↓ HF between 5–10 min after termination, ↔ LF, ↔ L/H ratio	• Pts with ischemic heart disease: ↓ RR interval from 15 min before until onset, ↑ SDNN, and ↑ rMSSD from 20 min before until onset; ↔ RR interval, ↓ SDNN, and ↓ rMSSD after termination • Pts without ischemic heart disease: ↔ RR interval, ↔ SDNN, ↑ rMSSD between 15–10 min before onset, ↑ RR interval from 5–10 min after termination, ↓ SDNN, and ↓ rMSSD after termination	-	• Pts with ischemic heart disease: ↑ sympathetic and parasympathetic activation before onset, ↓ parasympathetic activation for termination • Pts without ischemic heart disease: ↑ parasympathetic activation before onset, ↓ parasympathetic activation for termination
Sagnard et al., J Clin Med. 2020	Pts with acute myocardial infarction developing AF vs developing no AF	24-h period directly after hospital admission for acute myocardial infarction	↑ HR, ↓ L/H ratio	↔ SDNN, ↑ pNN50, ↑ rMSSD	-	↑ Parasympathetic and ↓ sympathetic
Zhong et al., Med Sci Monit. 2022	Pts with PAF vs controls • Sympathetic group • Vagal group	24-h period including time points 60, 45, 30, 15 min before and at PAF onset	• Overall: ↑ HF, ↑ LF • Sympathetic: ↔ HF, ↔ LF, ↔ L/H ratio over time, ↑ L/H ratio vs controls 15–0 min before onset • Vagal: ↓ HF from 60–45 min before onset, ↑ HF 45–0 min before onset, ↔ LF, ↓ L/H ratio over time, ↓ L/H ratio vs controls 15–0 min before onset	• Overall: ↑ SDNN 15 min before and at onset vs controls, ↑ pNN50 at onset, ↑ rMSSD • Sympathetic: ↔ SDNN, ↔ pNN50, ↔ rMSSD • Vagal: ↑ SDNN, ↓ pNN50, and ↓ rMSSD 60–45 min before onset, ↑ pNN50, and ↑ rMSSD 45–0 min before onset	-	↑ Parasympathetic and ↓ sympathetic • Vagal: ↓ parasympathetic → ↑ parasympathetic

*AF*, atrial fibrillation; *ApEn*, approximate entropy; *DFA1*, the short-term fractal scaling exponent of detrended fluctuation analysis; *HF*, high-frequency power; *HR*, heart rate; *HRV*, heart rate variability; *LF*, low-frequency power; *norm*, normalized; *L/H ratio*, ratio of low-frequency and high-frequency power; *PAF*, paroxysmal atrial fibrillation; *pts*, patients; *pNN50*, proportion of adjacent NN intervals differing > 50 ms; *rMSSD*, root-mean-square of differences between successive NN intervals; *SampEn*, sample entropy; *SDANN*, standard deviation of 5-min means of NN intervals; *SDNN*, standard deviation of NN intervals; *SD1RR*, average deviation of the plot data away from the long axis (axis 1); *SD2RR*, average deviation of the plot data away from the axis perpendicular to the long axis (axis 2); *SD/RR*, variation index; *VLF*, very low frequency

Despite the here introduced promising approach providing for the first time insights into cardiac autonomic function by HRV analysis in patients with PAF and OSA, certain methodological challenges should be kept in mind. First, HRV reflects “global” cardiac autonomic control by quantifying sinus nodal activity/control. To which extent these data are related to “local” intracardiac neural control on the atrial and ventricular level is not well understood. Furthermore, considering inclusion of unselected patients, sinus node dysfunction, which is often associated with AF, might have been a confounder in the here presented study. Despite recent developments in terms of additional nonlinear analyses, HRV remains a scientifically well studied but indirect measure of the cardiac autonomic nervous system, still leaving some questions unanswered.

Time- and frequency-domain HRV has recently demonstrated reliability across stable stage-2, slow-wave, and rapid eye movement sleep, while still remaining reliable during disrupted sleep [10]. In the here presented study, HRV analysis was performed during “steady-state” sinus rhythm excluding all OSA events and the immediate post-apneic period in order to compare chronic, but not acute, autonomic changes. However, these excluded time periods might give additional mechanistic insights into the underlying autonomic activation potentially explaining the oftentimes observed coexistence of AF and OSA.

In line with previous work suggesting OSA as an independent risk factor for AF [1], Mohammadieh et al. describe that PAF patients with severe OSA (apnea hypopnea index ≥ 30/h) had more AF beats than those without severe OSA as well as a higher AF burden than patients with moderate or without OSA. This observation confirms prior analyses indicating a strong association between OSA and AF and therefore suggesting a combined treatment of both comorbidities. Patients with OSA have a 1.7-fold increased risk of recurrent AF after catheter ablation compared to those without, and effective treatment of OSA using continuous positive airway pressure has been correlated with prolonged maintenance of sinus rhythm, with a 70% risk reduction of recurrent AF after catheter ablation [11]. Taken together, considering the present data, additional mechanistic studies as well as prospectively, randomized controlled trials are warranted to understand therapeutical options of interference with the autonomic nervous system as a fascinating piece of the mechanistic puzzle linking sleep and AF.
